# Comparative analysis of infection-associated and non-infection-associated pulmonary embolism in children: a multicenter retrospective cohort study

**DOI:** 10.3389/fped.2026.1858985

**Published:** 2026-08-13

**Authors:** Fengqin Liu, Sixian Liu, Lejia Zhang, Zhilang Lin, Jing Zhang, Huijuan Xu, Bing Liu, Tian Shen, Yuan Wen, Mengze Hu, Lirong Sun, Gang Liu, Xiaochuan Wu, Yi Zhang, Rong Liu, Xiaoyun Jiang, Xing Chen, Juan Xiao

**Affiliations:** 1 Pediatric Respiratory Department, Shandong Provincial Hospital Affiliated to Shandong First Medical University, Jinan, China; 2 Department of Pediatrics, Peking Union Medical College Hospital, Chinese Academy of Medical Sciences and Peking Union Medical College, Beijing, China; 3 Department of Pediatric Nephrology and Rheumatology, the First Affiliated Hospital, Sun Yat-sen University, Guangzhou, China; 4 Department of Pediatrics, The Affiliated Hospital of Qingdao University, Qingdao, China; 5 Department of Infectious Diseases, Beijing Children’s Hospital, Capital Medical University, Beijing, China; 6 Department of Pediatrics, the Second Xiangya Hospital, Central South University, Changsha, China; 7 Department of Pediatrics, Beijing Tongren Hospital, Capital Medical University, Beijing, China; 8 Department of Hematology, Children’s Hospital Capital Institute of Pediatrics, Beijing, China

**Keywords:** cardiopulmonary function, multicenter study, mycoplasma pneumoniae, pediatric pulmonary embolism, respiratory infection

## Abstract

**Background:**

Pediatric pulmonary embolism (PE) exhibits distinct risk factors, with respiratory infections playing a predominant yet understudied role in children.

**Methods:**

This multicenter, retrospective study analyzed pediatric patients with PE from 8 Chinese tertiary hospitals between 2003 and 2023. Patients were stratified into infection-associated PE (I-PE) and non-infection-associated PE (NI-PE) groups based on systemic infection status for comparative analysis.

**Results:**

Among 196 pediatric patients diagnosed with PE, I-PE was predominant at 75.5%, vs. 24.5% for NI-PE. The vast majority of I-PE group (77.0%) were associated with respiratory infections, among which 20.3% were attributable to *Mycoplasma pneumoniae*. Clinically, the I-PE group had higher inflammatory markers (CRP: 45.1 mg/L, ESR: 41 mm/h; *P* < 0.05) and more frequent chest pain (31.8% vs. 16.7%) and hemoptysis (19.6% vs. 0%; *P* < 0.05 for both). Low-risk stratification predominated in both groups (91.2% vs. 89.6%). Anticoagulation safety profiles were comparable (bleeding events: 5.4% vs. 4.2%), while the I-PE group without comorbidities demonstrated faster resolution of emboli (44 vs. 345 days). The PE-related mortality rate was 2.6% (below expected), with 10 (5.1%) cases developing late cardiopulmonary dysfunction.

**Conclusions:**

This study revealed a predominance of school-age children within the identified PE cohort, whose actual prevalence exceeding prior estimates. The association between infection, particularly acute respiratory infection, and PE in this cohort supports consideration of PE in children with respiratory infection and concerning features. I-PE presents with marked inflammation, a higher proportion of markedly elevated D-dimer (≥5 *μ*g/mL), and typical symptoms (chest pain/hemoptysis), yet demonstrates better short-term outcomes. NI-PE correlates with underlying diseases and warrants long-term complication monitoring. Recognizing these differences is vital for enhancing outcomes in children with respiratory diseases. PE should be considered in children with severe pneumonia who present with disproportionate inflammation or chest pain.

## Introduction

Pulmonary embolism (PE), once considered rare in children, is now recognized as a life-threatening condition with increasing clinical significance. While historical data suggested an incidence of only 0.005%, the associated mortality remains alarmingly high (up to 26%) (1). Improved survival in children with chronic illnesses and advances of imaging techniques have led to increased awareness and detection of PE in the pediatric population. Existing literature primarily consists of small-sample studies or data from limited North American centers (2–4), leaving critical gaps in our understanding of infection-associated PE, particularly regarding the spectrum of identifiable pathogens.

The rising prevalence of *Mycoplasma pneumoniae*(MP) infections in China has recently been linked to increased number of children with PE (5–7), highlighting the urgent need to characterize pathogen-specific thrombotic mechanisms. Unlike adult PE, pediatric PE present unique diagnostic challenges due to distinct clinical characteristics and the current lack of evidence-based guidelines. This multicenter retrospective study aimed to compare clinical characteristics between infection-associated and non-infection-associated pediatric PE and establish an evidence-based framework to develop risk-stratified management guidelines.

## Methods

### Study design

**“**PEOPEL” is a multi-center retrospective cohort of pediatric PE from 8 tertiary care centers in China (1. Shandong Provincial Hospital Affiliated to Shandong First Medical University. 2. Peking Union Medical College Hospital. 3. The First Affiliated Hospital, Sun Yat-sen University. 4. the Affiliated Hospital of Qingdao University. 5. Beijing Children's Hospital, Capital Medical University. 6. The Second Xiangya Hospital of Central South University. 7. Beijing Tongren Hospital, Capital Medical University 8. Children's Hospital Capital Institute of Pediatrics). Children with PE aged 1–18 years old who met the inclusion criteria between January 1, 2003 and December 31, 2023 were enrolled in this cohort, excluding children with a history of recurrent PE at first visit. The whole study was conducted in strict adherence to ethical norms and standards set in the Declaration of Helsinki, with approvals granted by the Research Ethics Board of the Hospital and exemption from informed consent based on the retrospective nature of the study. No generative artificial intelligence tools or technologies were used in the writing of the manuscript.

### Patients and study variables

Concurrent systemic infection was defined as an infection diagnosed within 14 days prior to the index date of PE diagnosis, supported by both clinical presentation and either microbiological confirmation (e.g., positive respiratory pathogen test, blood culture, etc.) or elevated inflammatory markers. Based on the definition, patients were categorized into infection-associated PE (I-PE) and non-infection-associated PE (NI-PE) groups. Data on demographics, medical history, comorbidities, clinical presentation, laboratory parameters, treatment strategies, and clinical course were collected. Prognostic information was obtained via electronic medical records and telephone follow-up (minimum follow-up: 3 months). Missing data were handled using complete-case analysis.

The I-PE group included patients with confirmed infections involving multiple systems (respiratory, gastrointestinal, hematologic, neurologic, urinary, skin/soft tissue, or osteomyelitis). The NI-PE group had no evidence of infection.

### Study definitions

According to the 2019 ESC Guidelines (8), risk stratification is determined by assessing haemodynamic status, clinical presentation, right ventricular function, and cardiac troponin levels. PE is classified as high-, intermediate-, or low-risk based on defined clinical and biochemical criteria (8). It should be noted that this risk stratification scheme, while widely used in adult and pediatric PE studies, has not been formally validated in pediatric patients. Therapeutic management was stratified into initial therapy (thrombolysis or anticoagulation) and sequential therapy (tailored to individual response). Pulmonary function tests adhered to 2021 ERS/ATS standards and Chinese pediatric guidelines (9, 10). Primary outcomes included: (1) clinical endpoints: all-cause and PE-related mortality, bleeding events, and cardiopulmonary functional deterioration; (2) radiographic endpoints: complete resolution (total thrombus disappearance), partial resolution (>50% reduction in thrombus burden with residual occlusion on CTPA), or progression/recurrence (new thrombi or >25% increase in thrombus burden on CTPA); (3) composite endpoints: incorporating both objective measures (documented recurrence, mortality) and functional outcomes (cardiopulmonary impairment, CTEPH).

### Statistical analysis

Continuous variables with normal distribution are presented as mean ± standard deviation (SD), while non-normally distributed variables are expressed as median [interquartile range (IQR), P25–P75]. Categorical variables are summarized as frequencies and percentages, with comparisons made using the chi-square test or Fisher's exact test, as appropriate. Multivariate logistic regression analysis was performed to adjust for potential confounders. All tests were two-sided, with *P* < 0.05 considered statistically significant. Missing outcome data were not imputed. Analyses were conducted using SPSS (25).

## Results

### General information

A total of 232 children with PE were initially enrolled. Thirty-six cases with more than 50% missing follow-up data were excluded. The final study cohort comprised 196 pediatric patients with PE, with a male predominance (105 males, 53.6%; 91 females, 46.4%; ratio 1.15:1), including 193 Han Chinese children (98.5%) and 3 Manchu children from 24 provinces across China. The average age of the patients was 10.41 ± 4.65 years, and the 6–14 year age group showed the highest incidence (62.8%). A total of 67 children required ICU care. The average hospital stay was 23.58 ± 18.78 days. Between 2018 and 2022, annual case numbers demonstrated an upward trend, consistently maintaining more than 20 cases per year with a peak incidence of 39 cases in 2021. In the I-PE group, 40.5% (*n* = 60) had comorbidities: autoimmune (*n* = 28), hematologic (*n* = 11), malignancies (*n* = 9), cardiac (*n* = 6), neurologic (*n* = 3), thrombophilia (Primary S protein deficiency, PSD = 2, Primary C protein deficiency, PCD = 1). The remaining 59.5% (*n* = 88) cases were patients with I-PE who had no comorbidities. In the NI-PE group, 52.1% (*n* = 25) with surgical/traumatic history, while 47.9% (*n* = 23) with comorbidities: malignancies (*n* = 11), autoimmune (*n* = 8), hematologic (*n* = 3), diabetes (*n* = 1). The proportion of missing values for baseline characteristics, laboratory parameters, and outcomes was <5%.

### Risk stratification assessment

A high proportion of patients in both the I-PE (135/148, 91.2%) and NI-PE (43/48, 89.6%) groups were classified as having a low-risk status. The database included a total of 18 medium- and high-risk cases, with low-risk cases significantly outnumbering medium- and high-risk cases. The proportion of medium-risk cases was 5.4% (8 cases) in the I-PE group and 8.3% (4 cases) in the NI-PE group.

High-risk cases were identified in 3.4% (5/148) of the I-PE group. Among these, 3 patients had significant underlying comorbidities, including 1 hemophagocytic syndrome, 1 osteosarcoma, and 1 nephrotic syndrome.

Additionally, 1 case (2.1%) in the NI-PE group, involving a car accident injury, was classified as high-risk (Table 1).

**Table 1:** Risk stratification distribution of I-PE vs. NI-PE groups.

Risk stratification	I-PE(*n* = 148)	NI-PE(*n* = 48)	*p*
High	5	1	0.102
	NUD(2); HPS(1); OS(1); NS(1)	A/T	
Intermediate	8	4	0.248
	NUD(2); NS(3); HB(1); T-LBL (1); LM (1)	NUD(3); CHF(1)	
Low	135	43	<0.001

NS, nephrotic syndrome; HPS, hemophagocytic syndrome; OS, osteosarcoma; NUD, no underlying disease; A/T accident/truma; HB, hepatoblastoma; T-LBL, T cell lymphoblastic lymphoma; LM, lupus myocarditis; CHF, Cardiomyopathy with heart failure.

### Clinical manifestation

Dyspnea was assessed using the Borg CR10 scale (11). Most patients in both groups reported scores of 0–3 (no symptoms to mild-moderate dyspnea): 70.9% (105/148) in the I-PE group vs. 87.5% (42/48) in the NI-PE group. Severe dyspnea (scores 4–8) occurred in 23.6% (35/148) of I-PE group and 8.3% (4/48) of NI-PE group, while very severe/extreme dyspnea (scores 9–10) was rare [I-PE: 5.4% [8/148]; NI-PE: 4.2% [2/48]]. No significant intergroup difference was observed (*P* > 0.05).

Hemoptysis occurred exclusively in the I-PE group (19.6%, 29/148; *P* < 0.001). Chest pain was more frequent in I-PEgroup (31.8%, 47/148) than in NI-PE group(16.7%, 8/48; *P* < 0.05). (Table 2).

**Table 2:** Clinical character and laboratory examination of I-PE vs. NI-PE groups.

Variable	I-PE(*n* = 148)	NI-PE(*n* = 48)	*P*	OR (95% Cl)
Presentation				
Dyspnea grade (*n*%)			0.058	
0–3	105 (70.9%)	42 (87.5%)		
4–8	35 (23.6%)	4 (8.3%)		
9–10	8 (5.4%)	2 (4.2%)		
Hemoptysis(*n*%)	29 (19.6%)	0 (0%)	<0.001	1.111 (0.998, 1.240)
Chest pain(*n*%)	47 (31.8%)	8 (16.7%)	0.043	2.327 (1.010, 5.359)
Risk stratification (*n*%)				
High	5 (3.4%)	1 (2.1%)	0.102	
With underlyding disease	3 (2.0%)	0 (0%)		
Medium	8 (5.4%)	4 (8.3%)	0.248	
Low	135 (91.2%)	43 (89.6%)	0.699	
Embolic site				0.821 (0.312, 2.159)
MPA with brach	1 (0.7)	1 (2.1)		
Unilateral	55 (37.2%)	13 (27.1%)	<0.001	
Bilateral	41 (27.7%)	7 (14.6%)	<0.001	
Glucocorticoid use(≥7d)	52 (35.1%)	11 (23.0%)	0.437	0.806 (0.468, 1.388)
Laboratory examination				
D-dimer(ug/mL)	64.7 (5.1, 124)	36.5 (8.7, 64.2)	0.625	1 (0.998, 1.001)
≥5(*n*%)	60 (40.5%)	13 (27.1%)	<0.001	1.001 (1.000, 1.001)
2.5–5(*n*%)	40 (27.0%)	5 (10.4%)	0.049	0.467 (0.101, 2.157)
<2.5(*n*%)	48 (32.4%)	30 (62.5%)	0.213	0.571 (0.237, 1.378)
Fib (g/L)	3.68 (2.54–6.89)	2.45 (1.60–3.65)	0.003	1.619 (0.875, 2.021)
PLT(10^9^/L)	261 (169, 352)	226 (143.5, 320)	0.141	1.000 (0.996, 1.001)
CRP(mg/L)	45.1 (36.1, 54.1)	24.0 (9.4, 38.5)	0.049	0.988 (0.976, 1.000)
CRP ≥ 100 (*n*%)	20 (13.5%)	2 (4.2%)	0.537	1.001 (0.998, 1.936)
ESR(mm/h)	41 (19.8–67.0)	21(9.5–57.5)	0.048	1.006(0.963, 1.052)

### CT pulmonary angiography (CTPA) findings

Aortic embolism was identified in 4 children (I-PE: 3, 2.0%; NI-PE: 1, 2.1%). 2 cases showed isolated aortic involvement, while 2 cases had concurrent embolism in bilateral pulmonary arteries; all 4 cases were high-risk. Bilateral pulmonary embolism was more frequent in the I-PE group (42.9%, 41/96) than in the NI-PE group (38.1%, 7/18; *P* < 0.001). Emboli predominantly affected the right pulmonary artery (39.3%, 77/196) vs. the left (21.4%, 42/196; *P* < 0.05) in both groups. Lower lobe involvement (23.5%, 46/196) significantly exceeded upper lobe involvement (1.0%, 2/196; *P* < 0.05), with no isolated right upper lobe emboli observed.

### Significant laboratory parameters

Both groups exhibited elevated D-dimer levels, though with distinct distribution patterns. No significant difference was observed in mean values [I-PE: 64.7 *μ*g/mL [95% CI: 5.1–124] vs. NI-PE: 36.5 *μ*g/mL [8.7–64.2]; *P* > 0.05]. However, the I-PE group showed a higher proportion of markedly elevated D-dimer levels, with 40.5% of patients having values ≥5 *μ*g/mL compared to 27.1% in the NI-PE group (*P* < 0.001), along with a greater frequency of moderate elevations (2.5–5 *μ*g/mL; 18.9% vs. 10.4%, *P* < 0.001) (Table 2). Furthermore, fibrinogen levels were significantly higher in I-PE group[3.68 g/L [2.54–6.89] vs. 2.45 g/L [1.60–3.65], *P* < 0.05]. Inflammatory markers were also elevated in the I-PE group, including CRP [45.1 mg/L [36.1–54.1] vs. 24 mg/L [9.4–38.5], *P* < 0.05] and ESR [41 mm/h [19.8–67.0] vs. 9 mm/h [9.5–57.5], *P* < 0.05] (Table 2).

Multivariable regression analysis revealed several independent predictors distinguishing I-PE from NI-PE. Chest pain (OR: 2.33, 95% CI: 1.01–5.36), D-dimer levels ≥ 5 µg/mL (OR: 1.00, 95% CI: 1.00–1.00), and elevated fibrinogen (OR: 1.62, 95% CI: 0.88–2.02) were identified as significant predictors. Additionally, hemoptysis (OR: 1.11, 95% CI: 1.00–1.24), CRP ≥ 100 mg/L (OR: 1.00, 95% CI: 1.00–1.94), and increased ESR (OR: 1.01, 95% CI: 0.96–1.05) showed marginal associations with I-PE diagnosis (Table 2).

### Classification and etiology of infections

Respiratory infections predominated as the leading cause of I-PE (77.0%, 114/148), followed by bloodstream infections (7.4%, 11/148). Less frequent etiologies included: Gastrointestinal infections (5.4%, 8/148), Skin/soft tissue infections (4.1%, 6/148), Osteomyelitis (2.7%, 4/148), CNS infections (2.0%, 3/148), Urinary tract infections (1.4%, 2/148). Comprehensive diagnostic testing (including molecular assays, cultures, and NGS) identified MP as the predominant pathogen (20.3%, 30/148).

Pathogen analysis identified that viral pathogens were detected in 11.5% of cases, with Epstein–Barr virus (EBV; *n* = 6), SARS-CoV-2 (*n* = 4), adenovirus (*n* = 3), and rhinovirus (*n* = 3) being the most frequently identified. Influenza B was found in 1 case. Bacterial and fungal pathogens were also identified in 11.5% of cases, predominantly *Acinetobacter baumannii* (*n* = 5) and *Staphylococcus aureus* (*n* = 4), followed by *Pseudomonas aeruginosa* (*n* = 2), *Klebsiella pneumoniae* (*n* = 2), and *Candida albicans* (*n* = 2). *Aspergillus spp.* and *Pneumocystis jirovecii* were each detected in 1 case.

### Treatment strategies

#### Thrombolysis

Thrombolysis was administered to 23 patients (11.7%) in the overall cohort, with no significant difference in frequency between the I-PE (10.8%, 16/148) and NI-PE (14.6%, 7/48) groups (*P* > 0.05). Urokinase served as the predominant thrombolytic agent in both the I-PE (68.8%, 11/16) and NI-PE (57.1%, 4/7) cohorts. Alteplase was the second most frequently administered agent, used in 25.0% (4/16) of I-PE group and 42.9% (3/7) of NI-PEgroup. Additionally, one patient (6.2%) in the I-PE group received a recombinant tissue-type plasminogen activator derivative.

#### Anticoagulant therapy and bleeding events

Due to the extended study period (2003–2023), anticoagulation regimens showed significant temporal variation in these cases. Bleeding complications occurred in 6.6% of patients (13/196), with comparable rates between I-PE (6.6%, 10/148) and NI-PE (6.3%, 3/48) groups (*P* > 0.05; Table 3).

**Table 3:** Anticoagulant therapy and bleeding events in I-PE vs. NI-PE groups.

Anticoagulant	I-PE(*n* = 148)		NI-PE(*n* = 48)		*P*
	Initial	Continuation	Initial	Continuation	
LMWH	81	22	26	6	—
Heparin	38	3	14	0	—
Rivaroxaban	17	31	4	6	—
Warfarin	12	21	4	12	—
Argatroban	0	1	0	1	—
Mild bleeding	5		1		0.102
Moderate bleeding	2		1		0.317
Severe bleeding	3		1		0.564
Total bleeding events	10		3		0.052

#### Prognosis of pulmonary embolism

This section reports the key outcome measures assessed through follow-up, including imaging resolution, mortality, and cardiopulmonary status.

### Imaging outcomes

During the follow-up period, 148 children had at least one CT scan that provided information on changes in emboli, with varying times for repeat CT scans at each center and department. Imaging follow-up demonstrated (1) Complete resolution: 81% (119/148) at median 4.8 months (IQR: 2.0–7.2); Partial resolution: 16% (24/148); Recurrence/progression: 3.4% (5/148). (2) Resolution kinetics in I-PE group: Partial resolution, 44 days (IQR: 20–46); Complete resolution: 73 days (IQR: 20–76). (3) Progression/recurrence: Overall, 2.6% (5/196); I-PE group, 2.0% (3/148; 1 protein S deficiency, 2 autoimmune diseases); NI-PE group, 4.2% (2/48; 1 hepatoblastoma with pulmonary metastases, 1 nephrotic syndrome) (*P* = 0.03).

Imaging follow-up showed complete resolution in 81% (119/148) of patients at a median of 4.8 months (IQR: 2.0–7.2), partial resolution in 16% (24/148), and progression/recurrence in 3.4% (5/148). In the I-PE group, median time to partial resolution was 44 days (IQR: 20–46) and to complete resolution 73 days (IQR: 20–76). Overall progression/recurrence rate was 2.6% (5/196), with significantly fewer cases in I-PE (2.0%, 3/148) than NI-PE (4.2%, 2/48; *P* = 0.03). Progressive I-PE group involved one protein S deficiency and two autoimmune diseases, while the NI-PE group included one hepatoblastoma with pulmonary metastases and one nephrotic syndrome.

### Mortality outcomes

During follow-up, all-cause mortality occurred in 5.6% of the cohort. PE-associated mortality accounted for 2.6% of all cases, with comparable rates between the I-PE (2.7%, 4/148) and NI-PE (2.1%, 1/48) groups (*P* > 0.05). All patients who died from PE were classified as high-risk, including two patients with systemic lupus erythematosus and two with severe respiratory infections in the I-PE group, and one trauma-associated case in the NI-PE group. The I-PE group also experienced four additional deaths from non-PE causes: two intermediate-risk patients (one with hepatoblastoma and one with uncomplicated infection) and two low-risk patients (one with severe Streptococcus pneumoniae infection and one with *Mycoplasma pneumoniae* pneumonia). The NI-PE group had two low-risk hepatoblastoma-associated deaths. All non-PE mortality cases were attributed to cardiac insufficiency and refractory disseminated intravascular coagulation. (Table 4).

**Table 4:** Prognosis of children with pulmonary embolism in I-PE vs. NI-PE groups.

Prognosis	I- PE(*n* = 148)		NI-PE(*n* = 48)	*P*
	with underlying condition	without underlying condition		
PE associated death	2	2	1	0.564
Other-cause death	1	3	2	0.414
Total	3	5	3	

### Cardiopulmonary outcomes

Four cases of fatal heart failure were documented: two patients in I-PE gropu with pre-existing comorbidities and two patients in NI-PE group. No instances of CTEPH were observed during the study period. Given the retrospective multicenter design, pulmonary function testing protocols were not standardized across participating centers. Among the patients who underwent testing, six cases of ventilatory dysfunction were identified: two with restrictive patterns and four with obstructive patterns. Notably, two of the patients with obstructive dysfunction were subsequently diagnosed with asthma.

## Discussion

The incidence of pediatric PE has risen markedly over the past two decades in both community and hospital settings (3, 12)., with diagnostic delays ranging from 0 to 42 days reported in prior studies (12). Our multicenter cohort study—the first and largest to characterize pediatric PE in China—reveals distinct epidemiological and clinical features that contrast sharply with adult PE presentations. These findings provide crucial insights into this frequently underrecognized condition in the pediatric population.

This study revealed a predominance of school-age children with PE, with a mean age of onset (10.41 ± 4.65 years) that was lower than previously reported. Earlier studies identified a peak in thrombosis incidence during adolescence—a population with adult-like characteristicss (1, 13). The study exhibited a male predominance (1.15:1), contrasting with the female predominance often observed in adolescents, which may be linked to combined hormonal contraceptive use (12). This demographic pattern likely reflects the absence of hormonal contraceptive exposure in our cohort (none reported among 91 female patients) and suggests age-specific risk factor profiles, which better reflects younger paediatric cases, distinguishing our study from prior reports. This demographic pattern likely reflects both the absence of hormonal contraceptive exposure (none reported among 91 female patients) and distinct, age-specific risk factor profiles.

Clinically, most children in both groups presented with asymptomatic to mild/moderate dyspnea (70.9% I-PE vs. 87.5% NI-PE; *P* > 0.05), though severe-to-critical respiratory distress occurred more frequently in the I-PE group (29.1% vs. 12.5% in NI-PE). While infection may worsen dyspnea in PE, overlapping symptoms (e.g., fever, cough) often obscure the diagnosis. Significantly, hemoptysis (19.6% vs. 0%; *P* < 0.001) and chest pain (31.8% vs. 16.7%; *P* < 0.05) were more common in I-PEgroup. The absence of classic PE triad symptoms in all patients underscores diagnostic challenges in children, attributable to both limited symptom articulation and remarkable cardiopulmonary reserve. Despite recruitment from eight major Chinese medical centres, underdiagnosis remains probable, reflecting either clinically silent presentations or frequent misdiagnosis of pediatric PE.

It is noteworthy that patients in I-PE groupexhibited significantly greater susceptibility to bilateral pulmonary embolism than patients in NI-PE group(*P* < 0.001), paralleling elevated D-dimer levels. PE predominance in the right lung in both groups aligns with the anatomical predilection of the right pulmonary artery (14).

In the present study, the observed D-dimer elevation (≥5 *μ*g/mL in 40.5% I-PE vs. 20.7% NI-PE, *P* < 0.01) reflects infection-associated hypercoagulability, mirroring reports in COVID-19 related VTE (15). D-dimer level was significantly higher in severe CAP patients than non-severe CAP patients, and was significantly elevated in non-survivors compared to survivors with CAP (16). However, D-dimer was not an independent predictor in the multivariate analysis after adjusting for CRP and fibrinogen, suggesting its elevation is driven by systemic inflammation rather than a specific thrombotic process. Therefore, clinicians should interpret elevated D-dimer levels in children with concurrent infection with caution.

The striking predominance of acquired risk factors (77.0% I-PE group, predominantly MP,) and temporal association in previously healthy children during epidemic peaks in China (2018–2022) (6, 7, 17), suggest acute infection represents a critical precipitant. Acute infections conferred an 11-fold higher PE risk within two weeks post-infection (18), likely mediated by infection-induced endothelial dysfunction and inflammatory activation of coagulation (19, 20). In this process, elevated inflammatory biomarkers (CRP, ESR) reflect an active systemic inflammatory state that drives hypercoagulation. Pathogen infections trigger intense immune responses, releasing inflammatory cytokines including IL-6 and TNF-α (21, 22). IL-6 not only stimulates the hepatic synthesis of acute-phase proteins like CRP but also upregulates tissue factor expression on monocytes and endothelial cells, initiating the extrinsic coagulation pathway. Additionally, inflammation induces endothelial activation and injury, promoting von Willebrand factor release and *P*-selectin expression, which facilitate leukocyte-platelet adhesion and thrombus formation (23). During infection, pathogens and their toxins activate thrombin generation, fibrin formation, and fibrinolysis, leading to elevated D-dimer levels that demonstrate significant correlations with both inflammatory markers (CRP, IL-6) and coagulation parameters (PT, APTT) (16).

Multivariate analysis identified hemoptysis, chest pain, D-dimer ≥ 5 *μ*g/mL, and elevated inflammatory markers (CRP, fibrinogen, ESR) as independent I-PE risk factors (Table 2). Worth noting is that D-dimer levels failed to predict disease severity in both I-PE and NI-PE groups, underscoring the critical need for pediatric-specific diagnostic algorithms. Radiographic and laboratory findings consistently demonstrated infection-induced hypercoagulability, manifested through multifocal pulmonary lesions and systemic inflammatory responses. These results establish acute respiratory infection as a critical trigger for pediatric PE, with our multicenter data revealing acquired risk factors to be substantially more prevalent than genetic predispositions. Importantly, the frequent symptom overlap between PE and pneumonia (particularly dyspnea and chest pain) necessitates heightened clinical vigilance to prevent diagnostic delays in this vulnerable population.

Conventional anticoagulants (heparin, LMWH, VKAs) remained the primary treatment for both I-PE and NI-PE in this cohort, with comparable efficacy observed between groups (Table 3). The absence of recurrence in I-PE group without underlying comorbidities reinforces evidence supporting limited-duration (3-month) therapy for transiently provoked VTE (24). While DOACs emerge as promising alternatives globally (24, 25), their off-label status in China underscores the urgent need for pediatric-specific formulations (e.g., weight-adjusted doses, liquid preparations) and pharmacologic data to optimize safety and adherence, particularly in young children.

These clinical observations correlate with risk stratification outcomes. Risk stratification revealed most cases (91.2% I-PE, 89.6% NI-PE) were low-risk, with overall mortality (2.6%) lower than historical reports (1). A Chinese multicenter adult PE study demonstrated decreasing in-hospital mortality from 1.4% (2009) to 0.5% (2015) (26), suggesting both the study period and patient age are critical determinants of outcomes. Current understanding of long-term outcomes following pediatric PE remains limited, with reported recurrence rates of 5%–18% in small series (27–29). While CTEPH and persistent dyspnea have been documented in children, their true incidence remains unknown (1). In the study, PE recurrence/progression occurred in 2.6% of cases, exclusively among children with underlying comorbidities in both I-PE and NI-PE groups. Of note, four patients in NI-PE groupdeveloped fatal cardiac insufficiency during follow-up, while no CTEPH cases were observed. These findings suggest a more favorable prognosis for patients in I-PE groupcompared to those inNI-PE group.

However, Emerging evidence highlights significant pulmonary sequelae post-PE in children, including persistent lung function impairment (2). The extended follow-up identified ventilatory dysfunction in six patients, with two patients (previously healthy) in I-PEgroup() developing bronchial asthma, suggesting the need for ongoing respiratory monitoring despite low recurrence rates. While a potential link between PE and subsequent asthma warrants further investigation, our data remain inconclusive due to limited sample size.

While pediatric PE has been historically underrecognized and our study did not include neonates (0–1 year), this multicenter study represents the largest pediatric PE cohort reported to date, revealing a significantly higher prevalence than previously estimated.

In conclusion, this comprehensive analysis identifies infection as a major associated condition in pediatric PE, with distinct pathophysiological and clinical characteristics differentiating I-PE from NI-PE. Key findings reveal that while I-PE exhibits more marked inflammatory responses and D-dimer elevation, it paradoxically demonstrates better short-term outcomes compared to NI-PE, which shows stronger comorbidity associations and greater long-term cardiopulmonary dysfunction risks. These findings support: (1) a lower threshold for considering PE while avoiding overtreatment in low-risk pediatric cases, pending future studies evaluating standard metrics (e.g., sensitivity, specificity) before recommending a diagnostic algorithm. (2) the development of age-appropriate risk stratification to optimize management protocols, and (3) the importance of structured long-term follow-up, particularly for NI-PE patients, to monitor and manage potential late cardiopulmonary complications.

Several limitations should be acknowledged. First, both the I-PE and NI-PE groups were heterogeneous regarding underlying etiologies. The relatively small sample size, particularly in the NI-PE group (*n* = 48), precluded robust multivariable adjustment for potential confounders. Second, given the exploratory nature of this study, we did not adjust for multiple comparisons, which may increase the risk of type I errors. Therefore, our findings should be interpreted as hypothesis-generating rather than confirmatory. Third, the 20-year study period may introduce temporal heterogeneity in clinical practice. Fourth, no formalsensitivity analyses (e.g., multiple imputation) were conducted to assess the impact of missing data on our results.

Fifth, our definition of radiographic endpoints relied on specific thresholds for resolution and progression, without an explicit category for stable disease (i.e., residual thrombus with neither significant regression nor progression). While this did not affect the classification of patients in our cohort who showed distinct trends, future prospective studies should include a stable category to ensure a more comprehensive classification framework. Despite these limitations, the absence of recurrence in I-PE patients without comorbidities (*n* = 88) qualitatively supports the robustness of our main conclusions. Future large-scale prospective studies are needed to validate these findings, particularly within pediatric populations.

## Data Availability

The raw data supporting the conclusions of this article will be made available by the authors, without undue reservation.

## References

[1] Rajpurkar M Biss TT Amankwah EK Martinez D Williams S Van Ommen CH. Pulmonary embolism and *in situ* pulmonary artery thrombosis in paediatrics. A systematic review. Thromb Haemost. (2017) 117:1199–207. 10.1160/TH16-07-0529 28331932

[2] Bastas D Brandão LR Vincelli J Wilson D Perrem L Guerra V. Long-term outcomes of pulmonary embolism in children and adolescents. Blood. (2024) 143:631–40. 10.1182/blood.2023021953 38134357

[3] Pelland-Marcotte M-C Tucker C Klaassen A Avila ML Amid A Amiri N. Outcomes and risk factors of massive and submassive pulmonary embolism in children: a retrospective cohort study. Lancet Haematol. (2019) 6:e144–53. 10.1016/S2352-3026(18)30224-2 30772417

[4] Rajpurkar M Huang YV Raffini L. Additional analysis of pediatric pulmonary embolism using the pediatric health information system database. Blood Adv. (2019) 3:2604–7. 10.1182/bloodadvances.2019000071 31488437 PMC6737405

[5] Chen L Yin J Liu X Liu J Xu B Shen K. Thromboembolic complications of *Mycoplasma pneumoniae* pneumonia in children. Clin Respir J. (2023) 17:187–96. 10.1111/crj.13584 36658687 PMC9978901

[6] Song S Xu Y. A retrospective study of the clinical characteristics of 9 children with pulmonary embolism associated with Mycoplasma pneumoniae pneumonia. BMC Pediatr. (2023) 23:370. 10.1186/s12887-023-04188-7 37474910 PMC10360226

[7] Liu F Zhang J Chen X Ding N Dai F Wang K. Clinical analysis and etiology factor of pulmonary embolism in 30 children. Chin J Appl Clin Pedia. (2022) 37:1386–91. 10.3760/cma.j.cn101070-20220126-00099

[8] Konstantinides SV Meyer G Becattini C Bueno H Geersing G-J Harjola V-P. 2019 ESC guidelines for the diagnosis and management of acute pulmonary embolism developed in collaboration with the European respiratory society (ERS): the task force for the diagnosis and management of acute pulmonary embolism of the European Society of Cardiology (ESC). Eur Respir J. (2019) 54:1901647. 10.1183/13993003.01647-2019 31473594

[9] Stanojevic S Kaminsky DA Miller MR Thompson B Aliverti A Barjaktarevic I. ERS/ATS technical standard on interpretive strategies for routine lung function tests. Eur Respir J. (2022) 60:2101499. 10.1183/13993003.01499-2021 34949706

[10] Respiratory Group of Pediatric Society, Chinese Medical Association & Pulmonary Function Cooperative Group, Editorial Board of Chinese Journal of Applied Clinical Pediatrics. Series guidelines for pediatric pulmonary function testing (part 2): lung volumes and ventilatory function. Chin J Appl Clin Pedia. (2016) 31:744–50. 10.3760/cma.j.issn.2095-428X.2016.10.006

[11] Borg GA. Psychophysical bases of perceived exertion. Med Sci Sports Exerc. (1982) 14(5):377–81. 10.1249/00005768-198205000-00012 7154893

[12] Lopes de Bragança R Gorito V Cibele DG Ricca Gonçalves L Ribeiro A Baptista MJ. Pulmonary embolism in pediatric age: a retrospective study from a tertiary center. Pediatr Pulmonol. (2021) 56:2751–60. 10.1002/ppul.25527 34133850

[13] Biss TT. Pulmonary embolism in childhood: how can we be sure not to miss it. Arch Dis Child. (2018) 103:814–6. 10.1136/archdischild-2017-314428 29550763

[14] Tapson VF. Acute pulmonary embolism. N Engl J Med. (2008) 358:1037–52. 10.1056/NEJMra072753 18322285

[15] Righini M Van Es J Den Exter PL Roy P-M Verschuren F Ghuysen A. Age-adjusted D-dimer cutoff levels to rule out pulmonary embolism: the ADJUST-PE study. JAMA. (2014) 311:1117–24. 10.1001/jama.2014.2135 24643601

[16] Li J Zhou K Duan H Yue P Zheng X Liu L. Value of D-dimer in predicting various clinical outcomes following community-acquired pneumonia: a network meta-analysis. PLoS One. (2022) 17:e0263215. 10.1371/journal.pone.0263215 35196337 PMC8865637

[17] Zhang J Wu R Mo L Ding J Huang K. Trends in *Mycoplasma pneumoniae* infections in pediatric patient preceding, during, and following the COVID-19 pandemic: a comprehensive longitudinal analysis. Microbiol Spectr. (2025) 13:e0100124. 10.1128/spectrum.01001-24 39807865 PMC11792468

[18] Smeeth L Cook C Thomas S Hall AJ Hubbard R Vallance P. Risk of deep vein thrombosis and pulmonary embolism after acute infection in a community setting. Lancet. (2006) 367:1075–9. 10.1016/S0140-6736(06)68474-2 16581406

[19] Koster T Rosendaal FR Lieuw-A-Len DD Kroes AC Emmerich JD Van Dissel JT. Chlamydia pneumoniae IgG seropositivity and risk of deep-vein thrombosis. Lancet. (2000) 355:1694–5. 10.1016/S0140-6736(00)02244-3 10905248

[20] Lozinguez O Arnaud E Belec L Nicaud V Alhenc-Gelas M Fiessinger J-N. Demonstration of an association between Chlamydia pneumoniae infection and venous thromboembolic disease. Thromb Haemost. (2000) 83:887–91. 10.1055/s-0037-1613938 10896243

[21] Zhao J Li Y Zhang W. The clinical significance of IL-6s and IL-27s in bronchoalveolar lavage fluids from children with mycoplasma pneumoniae pneumonia. BMC Infect Dis. (2020) 20:331. 10.1186/s12879-020-05017-3 32393186 PMC7216321

[22] Garcia-Larragoiti N Cano-Mendez A Jimenez-Vega Y Trujillo M Guzman-Cancino P Ambriz-Murillo Y. Inflammatory and prothrombotic biomarkers contribute to the persistence of sequelae in recovered COVID-19 patients. Int J Mol Sci. (2023) 24:17468. 10.3390/ijms242417468 38139298 PMC10744310

[23] Galeano-Valle F Ordieres-Ortega L Oblitas CM Del-Toro-Cervera J Alvarez-Sala-Walther L Demelo-Rodríguez P. Inflammatory biomarkers in the short-term prognosis of venous thromboembolism: a narrative review. Int J Mol Sci. (2021) 22:2627. 10.3390/ijms22052627 33807848 PMC7961591

[24] Duffett L Castellucci LA Forgie MA. Pulmonary embolism: update on management and controversies. Br Med J. (2020) 370:m2177. 10.1136/bmj.m2177 32759284

[25] Ortel TL Neumann I Ageno W Beyth R Clark NP Cuker A. American society of hematology 2020 guidelines for management of venous thromboembolism: treatment of deep vein thrombosis and pulmonary embolism. Blood Adv. (2020) 4:4693–738. 10.1182/bloodadvances.2020001830 33007077 PMC7556153

[26] Zhai Z Wang D Lei J Yang Y Xu X Ji Y. Trends in risk stratification, in-hospital management and mortality of patients with acute pulmonary embolism: an analysis from the China pUlmonary thromboembolism REgistry study (CURES). Eur Respir J. (2021) 58:2002963. 10.1183/13993003.02963-2020 33986031

[27] Biss TT Brandão LR Kahr WH Chan AK Williams S. Clinical features and outcome of pulmonary embolism in children. Br J Haematol. (2008) 142:808–18. 10.1111/j.1365-2141.2008.07243.x 18564359

[28] Hancock HS Wang M Gist KM Gibson E Miyamoto SD Mourani PM. Cardiac findings and long-term thromboembolic outcomes following pulmonary embolism in children: a combined retrospective-prospective inception cohort study. Cardiol Young. (2013) 23:344–52. 10.1017/S1047951112001126 23088931

[29] Tavil B Kuskonmaz B Kiper N Cetin M Gumruk F Gurgey A. Pulmonary thromboembolism in childhood: a single-center experience from Turkey. Heart Lung. (2009) 38:56–65. 10.1016/j.hrtlng.2007.06.004 19150531

