# Supplementary material for: Comparative analysis of infection-associated and non-infection-associated pulmonary embolism in children: a multicenter retrospective cohort study
Source: Front Pediatr. 2026 Aug 13;14:1858985. doi: 10.3389/fped.2026.1858985 (PMC13518526; doi:10.3389/fped.2026.1858985)
Supplement: Supplementary file 1 [file Datasheet1.pdf]

The detailed reasons for PE in the non-infectious group were indeed already listed in the “General information”section on page 6: "In the NI-PE group, 52.1% (n=25) had a surgical/traumatic history, while 47.9% (n=23) had comorbidities: malignancies (n=11), autoimmune (n=8), hematologic (n=3), and diabetes (n=1)." Following the reviewer's suggestion, we have listed the 23 patients with comorbidities in the NI-PE group in the following table.

Supplementary Table 1. Detailed comorbidities of the 23 patients in the NI-PE group

patient	comorbidity
1	Nephroblastoma
2	Malignant Germ Cell Tumor
3	Lymphoma
4	Lymphoma
5	Neuroblastoma
6	Hepatoblastoma
7	Hepatoblastoma
8	Hepatoblastoma
9	Hepatoblastoma
10	Hepatoblastoma
11	Osteosarcoma
12	Nephrotic syndrome and Antiphospholipid syndrome
13	Systemic Lupus Erythematosus and Lupus Nephritis (nephrotic syndrome type)
14	Systemic Lupus Erythematosus and Lupus Nephritis (nephrotic syndrome type)
15	Behcet's disease and Nephrotic syndrome
16	Antiphospholipid syndrome
17	Nephrotic syndrome and Antiphospholipid syndrome
18	Systemic Lupus Erythematosus and Lupus Nephritis (nephrotic syndrome type)
19	Henoch-Schönlein Purpura and Henoch-Schönlein Purpura Nephritis (nephrotic syndrome type)
20	Acute Myeloid Leukemia
21	Acute Lymphoblastic Leukemia
22	T-cell acute lymphoblastic leukemia
23	Diabetes Mellitus
